# The impact of Metastasis Suppressor-1, MTSS1, on oesophageal squamous cell carcinoma and its clinical significance

**DOI:** 10.1186/1479-5876-9-95

**Published:** 2011-06-22

**Authors:** Fei Xie, Lin Ye, Jinfeng Chen, Nan Wu, Zhiqian Zhang, Yue Yang, Lijian Zhang, Wen G Jiang

**Affiliations:** 1 Metastasis and Angiogenesis Research Group, Cardiff University School of Medicine, Cardiff, CF14 4XN, UK; 2 Key Laboratory of Carcinogenesis and Translational Research (Ministry of Education), Department of Thoracic Surgery, Peking University School of Oncology and Beijing Cancer Hospital, Beijing, 100142, China

**Keywords:** metastasis suppressor-1, MTSS1, MIM, oesophageal squamous cell carcinoma, metastasis

## Abstract

**Background:**

Metastasis suppressor-1 (MTSS1) has been proposed to function as a cytoskeletal protein with a role in cancer metastasis. Recent studies have demonstrated the clinical significance of MTSS1 in certain type of cancers, yet the clinical relevance of MTSS1 in oesophageal squamous cell carcinoma (ESCC) has not been reported.

**Methods:**

In this study, we assessed the expression levels of MTSS1 in tumours and its matched adjacent non-tumour tissues obtained from 105 ESCC patients. We also used ESCC cells with differing MTSS1 expression and assessed the influence of MTSS1 on ESCC cells.

**Results:**

Down-regulation of MTSS1 expression was observed both in oesophageal tumour tissues and ESCC cancer cell lines. We also reported that MTSS1 expression was associated with tumour grade (p = 0.024), lymph node metastasis (p = 0.010) and overall survival (p = 0.035). Patients with high levels of MTSS1 transcripts had a favorable prognosis in comparison with those who had reduced or absent expression levels. Using over-expression and knockdown approach, we created sublines from ESCC cells and further demonstrated that MTSS1 expression in ESCC cells significantly influenced the aggressiveness of the oesophageal cancer cells, by reducing their cellular migration and in vitro invasiveness.

**Conclusion:**

MTSS1 serves as a potential prognostic indicator in human ESCC and may be an important target for cancer therapy.

## Background

Tumour metastasis is the most significant contributor to the mortality of patients with cancers. Metastasis of cancer cells proceeds via a long series of sequential, interrelated steps modulated largely by activators and suppressors of metastasis. Metastasis suppressor genes are defined by their ability to inhibit metastasis at any step of the metastatic cascade. To date, only a limited number of metastasis suppressor genes, including NM23, KAI1, KiSS1, MKK4, BRMS1, RHOGDI2, CRSP3 and VDUP1, have been identified [1]. These metastasis suppressor genes inhibit metastasis of a cancer cell line *in vivo *without blocking its tumourigenicity.

MTSS1 (metastasis suppressor-1), also known as MIM (Missing-In-Metastasis), MIM-B, BEG4 (Basal cell carcinoma-enriched gene 4) or KIAA0429, was first identified as a potential metastasis suppressor gene missing in metastatic bladder carcinoma cell lines [2] and subsequently investigated in some types of cancer. In prostate cancer and breast cancer, expression of MTSS1 has been shown to be reduced, whereas up-regulation of MTSS1 expression has also been observed in hepatocellular carcinoma [3]. MTSS1 may exert its metastasis suppressor functions by acting as a scaffold protein that interacts with actin-associated proteins to regulate lamellipodia formation [4-6]. Biochemical study revealed that MTSS1 binds monomeric actin through its C-terminal WH2 domain for polymerization and deforms phosphoinositide-rich membranes through its N-terminal I-BAR domain [6,7]. MTSS1 has also been identified as a sonic hedgehog inducible protein that potentiates Gli transcription in the developing hair follicle and basal cell carcinomas of the skin [8]. To date, the role and biochemical mechanisms for MTSS1 in tumourigenesis and metastasis remain largely unknown. This is due partly to the fact that the studies of MTSS1 have been restricted to a limited number of cancer types, with little support from the clinical aspect.

Until now, there has been no research reporting the role of MTSS1 in oesophageal squamous cell carcinoma (ESCC). Here, we sought to determine MTSS1 expression in oesophageal cancer patient specimens and evaluate the clinical implications of MTSS1 expression in oesophageal squamous cell carcinoma. We also provide new insights into the biological functions of MTSS1 and its role in oesophageal squamous cell carcinoma.

## Material and methods

### Cell lines and human oesophageal specimens

This study used three human oesophageal squamous cell carcinoma cell lines and an oesophageal adenocarcinoma cell line. Moderate-differentiated cell lines OE19 (oesophageal adenocarcinoma cell line) and OE21 were obtained from the European Collection for Animal Cell Culture (ECACC, Porton Down, Salisbury, UK). The other two oesophageal cancer cell lines KYSE150 (poorly-differentiated) and KYSE510 (well-differentiated) were gifted from Dr. Zhiqian Zhang (Beijing Institute for Cancer Research). Cells were routinely cultured with Dulbecco's modified Eagle medium (DMEM) supplemented with 10% foetal calf serum, penicillin and streptomycin (Gibco BRC, Paisley, Scotland, UK).

Fresh frozen oesophageal squamous cell carcinoma tissues (n = 105), along with matched normal tissue from the same patients, were obtained from patients who attended Beijing Cancer Hospital from January 2003 to December 2009. Ethical approval was provided by the Beijing Cancer Hospital Ethics Committee. None of the patients received any neoadjuvant therapy prior to surgery. Histological types of the oesophageal squamous cell carcinoma patients are given in table 1. These tissues were collected immediately after surgical resection at the Beijing Cancer Hospital and were stored in the Tissue Bank of Peking University Oncology School. Clinico-pathologic factors, including age, sex, histological types of tumours, TNM stage, and lymph node metastasis were recorded and stored in the patients' database. Patients were followed up from the day of operation to December 2009 as the end of the follow-up for the present study. The follow-up intervals were calculated as survival intervals after surgery.

**Table 1:** Clinical data of the patients with oesophageal squamous cell carcinoma.

Clinical data	Grouping	Sample number
Tissue Sample	Tumour	105
	Normal	105
Gender	Male	87
	Female	18
Age	> 55	64
	≤ 55	41
T status	1	1
	2	25
	3	56
	4	19
N status	0	48
	1/2	53
M status	0	99
	1	2
WHO grade	1	7
	2	46
	3	41
	4	7

### RNA isolation and reverse transcription polymerase chain reaction

Total RNA was isolated from the homogenized oesophageal tissues and cell lines using Total RNA Isolation Reagent (ABgene™). Reverse transcription was done using the Reverse Transcription kit (Primer design), followed by PCR using a REDTaq™ ReadyMix PCR reaction mix (Sigma-Aldrich, Inc.). RNA concentration was determined through spectrophotometric measurement (WPA UV 1101, Biotech Photometer, Cambridge, UK). 1 μg RNA was used to generate cDNA with a RT kit (AbGene Laboratories, Essex, England). The quality of DNA was verified using GAPDH primers (sense: 5'-ATGATATCGCCGCGCTCGTC-3'; antisense: 5'-CGCTCGGTGAGGATCTTCA-3'). MTSS1 mRNA levels were assessed using MTSS1 primers (sense: 5'-TCAAGAACAGATGGAAGAATGG-3'; Antisense: 5'-TGCGGTAGCGGTAATGTG-3', exon 5-10). PCR was performed in a GeneAmp PCR system 2400 thermocycler (Perkin-Elmer, Norwalk CT, USA). Cycling conditions for the 16-μl-reaction mixture were 30s at 94°C for denaturation, 30s at 55°C for annealing and 30s at 72°C for elongation (30 cycles). This was followed by a final 10 min extension period at 72°C. PCR products were then separated on a 2% agarose gel. The gel was then visualized under ultraviolet light following ethidium bromide staining.

### Quantitative real time PCR

Real time quantitative PCR (QPCR) was performed on the Icycler IQ5 system (Bio-Rad, Hammel Hemstead, UK) to quantify the level of MTSS1 transcripts in the oesophageal squamous cell carcinoma specimens (shown as copies/μl from internal standard). Oesophageal cDNA samples were then examined for MTSS1 transcript expression, along with a set of standards and negative controls [9]. The QPCR technique utilized the Amplifluor system™ (Intergen Inc., England) [10] and QPCR master mix (BioRad). Pairs of primers as follow were designed using Beacon Design software (PREMIER Biosoft, Palo Alto, CA): MTSS1 QPCR primers as follow: sense: 5'-ATATCCCAGGATGCCTTC-3'; antisense: 5'-ACTGAACCTGACCGTACACGGTTCTCGCTTCTCTTT-3', exon 10-12). The underlined sequence in the reverse primers was the additional Z sequence, which is complementary to the universal Z probe (TCS Biologicals Ltd., Oxford, UK). Real-time QPCR conditions were 95°C for 15 min, followed by 60 cycles at 95°C for 20 s, 55°C for 30 s and 72°C for 20 s. QPCR for GAPDH was also performed on the same samples to normalize for any residual differences in the initial level of RNA in the specimens, using a GAPDH quantitation kit from Perkin-Elmers (Perkin-Elmer, Surrey, England, UK).

### Immunohistochemical staining of MTSS1

Paraffin sections of oesophageal squamous cell carcinoma tumours (n = 35) and matched background tissues (n = 35) were cut at a thickness of 6 μm. The sections were first dewaxed using a series of zylene and ethanol washes. Endogenous peroxidase activity was blocked with 0.3% hydrogen peroxide for 15 min. For antigen retrieval, sections were boiled in 10 mM citrate buffer (pH 6.0) for 10 min. The sections were then immersed in 'Optimax' wash buffer for 10 min to rehydrate and incubated for 20 min in a horse serum blocking solution before probing with the MTSS1 antibody (1:80) (Abnova, Caltag-Med-systems Ltd., Buckingham, UK) and also without primary antibody as a negative control. Following extensive washing, sections were incubated for 30 min with the secondary biotinylated antibody (Multilink swine anti-goat/mouse/rabbit immunoglobulin, Dako Inc. Carpinteria, CA). Avidin-biotin complex (Vector Laboratories) was then applied to the sections followed by extensive washing. Diamino benzidine chromogen (Vector Laboratoriess) was then added to the sections and incubated in the dark for 5 min. Sections were then counterstained in Gill's haematoxylin and dehydrated in ascending grades of methanol before clearing in xylene and mounting under a cover slip. Staining was independently assessed by the authors.

### Construction of MTSS1 Expressing and Ribozyme Transgenes, and Transfection

The full sequence of MTSS1 was amplified from PLC/PRF-5 cDNA, using the standard PCR procedure described above and a master mix with proof reading enzyme, we previously reported [11,12]. The following primers which allowed amplification of the full length human MTSS1 (exon 1-14) were used: sense primers: 5'-ATGGAGGCTGTGATTGAG-3'; antisense: 5'-CTAAGAAAAGCGAGGGG-3'. Correctly amplified product was then cloned into pEF6/V5-His-TOPO vector (Invitrogen, Paisley, UK). Multiple clones of *E. coli *were screened and plasmids from the clones were sequenced. Detailed procedure was adapted from the reports described previously [12]. Purified plasmids were then electroporated into the KYSE150 oesophageal cancer cell line. Blasticidin (5 μg/ml final concentration) was used to select stably transfected strains. The KYSE150 cells stably expressing MTSS1 were termed in the study as KYSE150-MTSS1-Exp. The control group of cells contained the same plasmid vector (minus the MTSS1 sequence) and was termed KYSE150-PEF-control.

Anti-MTSS1 ribozyme transgenes were employed to knockdown the expression of MTSS1 in the KYSE510 oesophageal cancer cell line, and were generated using the methods previously described [12]. Briefly, the anti-MTSS1 hammerhead ribozyme targeting was designed based on the secondary structure generated using Zuker's RNA mFold program. Then the ribozymes that specifically target MTSS1 were generated using touchdown PCR with the appropriate primers (sense: 5'-CTGCAGAGGCTTTTTAGATCTTCCGACTGATGAGTCCGTGAGGA-3'; antisense: 5'-ACTAGTTAACCCACCTTCAGACCATTTCGTCCTCACGGACT-3'). Correctly amplified inserts were purified and cloned into the pEF6/V5-His-TOPO vector, before transfecting the KYSE510 oesophageal cancer cell line by way of electroporation. The same procedure as described above was employed to select stably transfected strains. KYSE510 cells with MTSS1 eliminated and the control group of cells contained the same plasmid vector were termed KYSE510-MTSS1-Rib and KYSE510-PEF-control respectively.

### Western blotting

To detect the expression level of MTSS1 in the oesophageal cancer cell lines, confluent cells were pelleted and then lysed using a lysis buffer containing 2.4 mg/ml Tris, 4.4 mg/ml NaCl, 5 mg/ml sodium deoxycholate, 20 μg/ml sodium azide, 1.5% Triton, 100 μg/ml PMSF, 1 μg/ml leupeptin, and 1 μg/ml aprotinin, for 45 min at 4°C. After lysis and centrifugation at 13,000 rpm for 15 min, protein concentrations for each sample were measured using an improved Lowary assay (DC Protein Assay kit, Bio-Rad). The samples were adjusted to equal concentrations with sample buffer and then boiled at 100°C for 5 min, before separated on a 10% polyacrylamide gel. Following electrophoresis, these separated proteins were blotted onto nitrocellulose sheets and blocked in 10% skimmed milk (w/v in TBS) for 1 hour. The membranes were then probed with the anti-MTSS1-antibody (Abnova, Caltag-Med-systems Ltd., Buckingham, UK) and anti-actin-antibody (Santa-Cruz Biotechnologies, California, USA) as internal control, followed by a peroxidase-conjugated secondary antibody (1:1,000). Protein bands were visualised using an ECL system (Amersham, UK), and photographed using a UVITech imager (UVITech, Inc.).

### *In vitro *cell growth assay

Cells were plated into 96-well plated at 2,000 cells/well after a period of incubation. Cells were fixed in 10% formaldehyde after 1, 3 and 5 days. 0.5% crystal violet (w/v) was used to stain cells. Following washing, the stained crystal violet was dissolved with 10% (v/v) acetic acid and the absorbance was determined at a wavelength of 540 nm using a spectrophotometer (Bio-Tek, ELx800).

### Cell matrix adhesion assay

The cell matrix adhesion assay was done as previously described [13]. 96-well plate was precoated with 5 μg of Matrigel and allowed to dry. Following rehydration, 30,000 cells were added to each well. After 40 min of incubation non-adherent cells were washed off using BSS buffer. The remaining cells were fixed with 4% formalin and stained with 0.5% crystal violet. The number of adherent cells was then counted under microscopy.

### Wounding/migration assay

The wounding assay was performed as previously described [14]. The cells were seeded at a density of 40,000 per well into a 24-well plate and allowed to reach confluence. The monolayer of cells was then scraped with a fine gauge needle to create a wound of approximately 200 μm. The movement of cells to close the wound was recorded as described previously using a time-lapsed video system. Images were captured from the videotape at the equivalent of 15 min intervals in real-time and stored as a series of gray scale bitmap images. The movement of single cells within a colony was analyzed by tracking each cells boundary, for each frame in a series, using the Optimas 6.0 motion analysis (Meyer Instruments, Houston, Texas).

### In vitro invasion assay

Transwell inserts (upper chamber) with 8 μm pore size were coated with 50 μg of Matrigel (Collaborative Research Products, Bedford, Massachusetts, USA) and air-dried. Following rehydration, cells were seeded at a density of 30,000 per insert and allowed to invade for 3 days. After incubation, cells that had migrated through the matrix and adhered to the other side of the insert were fixed in 4% formalin, stained with 0.5% (weight/volume) crystal violet, and counted under a microscope.

### 2.11 Statistical analysis

Statistical analysis was performed using SPSS software (SPSS Standard version 13.0, SPSS Inc.). The relationship between MTSS1 expression and tumour grade, TNM staging and nodal status was assessed by Mann-Whitney U test. The error bar shown in the graph represents the SEMs. Survival curve was analyzed using Kaplan-Meier survival analysis. Multivariate analysis of the impact of the factors, including gender, age, grade, TNM, nodal status and MTSS1 expression levels, were conducted using the Cox regression model. Differences were considered statistically significant at p < 0.05.

## Results

### Quantitative PCR analysis of the expression pattern of MTSS1 in oesophageal squamous cell carcinoma tissues

#### MTSS1 mRNA expression in oesophageal squamous cell carcinoma tissues

**Figure 1:**
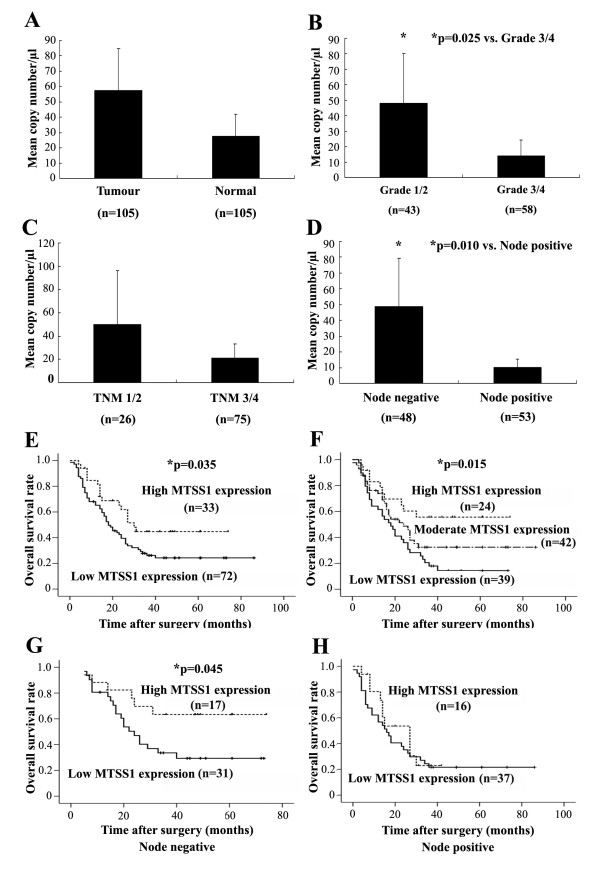
**Quantitative PCR analysis of MTSS1 expression in human oesophageal tissues**. (A) Tumour versus normal background tissues; (B) Tumour grade; (C) Tumour-node-metastasis classification; (D) Node status; (E) A two-way division of the patients based on the expression levels of MTSS1 yield a significant correlation with overall survival; (F) A three-way division of the patients based on the expression levels of MTSS1 yield a significant correlation with overall survival; (G) Overall survival analysis in node negative patients; (H) Overall survival analysis in node positive patient.

MTSS1 transcript expression was examined in the oesophageal specimens of 105 oesophageal squamous cell carcinoma patients using real-time quantitative PCR (expressed as mean MTSS1 transcript copies/μl of RNA from 50 ng total RNA and standardized with GAPDH). The cohort comprised 87 men (82.86%) and 18 women (17.14%). The average age of all patients was 58.75 years. Lower mRNA expression level of MTSS1 was observed in tumour tissues (27.42 ± 7.32) when compared to the normal background tissues (57.38 ± 13.61), although the difference was only marginally statistically significant (p = 0.054)(Figure 1A).

#### MTSS1 expression correlates with tumour grade or TNM staging

The relation of MTSS1 expression against pathological status was also assessed in the present study through quantitative analysis of MTSS1 transcript. Since the tumour grade and TNM staging information of 4 cases in the cohort was missing, the tissues used for the analysis of tumour grade, T status, N status were 101. MTSS1 levels were first assessed in relation to oesophageal tumour grade (grade 1, n = 6; grade 2, n = 46; grade 3, n = 41; grade 4, n = 7). Tumour grade (well or moderately differentiated (grade 1/2) vs. poorly differentiated or undifferentiated (grade 3/4)) outcomes are shown in Figure 1B. Grade 3/4 tumours (13.82 ± 5.13) had significant reduced levels of MTSS1 compared to grade 1/2 tumours (48.19 ± 16.07) (p = 0.024).

The relationship between MTSS1 expression and clinical TNM staging was also analyzed. Patient TNM grouping revealed that TNM 3/4 patients had lower expression levels of MTSS1 (20.99 ± 6.23) in comparison with the TNM 1/2 group (49.97 ± 23.24). However, statistical analysis show no significant difference (p = 0.095, Figure 1C).

#### MTSS1 expression in relation to nodal status

As lymph node metastasis is one of the most important prognostic factors, we next explored possible correlations between MTSS1 expression levels and lymph node metastasis. The patients were divided into two groups based on the nodal status: the first group included N0 patients and the second group N1/N2 patients. We found a significant correlation between MTSS1 expression level and nodal status (p = 0.010). Quantitative studies showed that MTSS1 expression levels in node positive patients were significantly lower (10.06 ± 2.65) than those without metastases (48.76 ± 15.27) (Figure 1D).

#### Correlation between MTSS1 expression and oesophageal squamous cell carcinoma patient survival

Of the 105 oesophageal squamous cell carcinoma patients, none was lost to follow-up. The median observation period was 20 months (0-86 months). Kaplan-Meier analysis demonstrated that patients with high levels of MTSS1 expression in their tumours showed a longer overall survival time (30.00 ± 3.70 months), compared with lower expression levels group (18.00 ± 2.78 months) (p = 0.035, Figure 1E). We further characterize the patients into three groups according to MTSS1 expression levels: high, moderate or low levels. Most remarkably, patients with high MTSS1 levels had the longest survival time (47.70 ± 6.32 months), compared with those with moderate (38.22 ± 5.41 months) or low MTSS1 levels (24.64 ± 3.61 months) (p = 0.015, Figure 1F). In stratified survival analysis according to the node status, node negative patients with high MTSS1 levels had a significant longer survival (53.41 ± 6.82 months) in comparison with low levels group (34.51 ± 4.79 months) (p = 0.045, Figure 1G). In the node positive patients, no significant association was found between MTSS1 expression and survival (p > 0.05, Figure 1H). Finally, multivariate analysis using gender, age, grade, TNM, nodal status and MTSS1 expression levels as variants has shown that nodal status (p = 0.015), TNM (p = 0.006), grade (p = 0.026), age (p = 0.020) and MTSS1 (p = 0.037) are independent factors for the overall survival.

### Immunohistochemical staining of human oesophageal specimens

**Figure 2:**
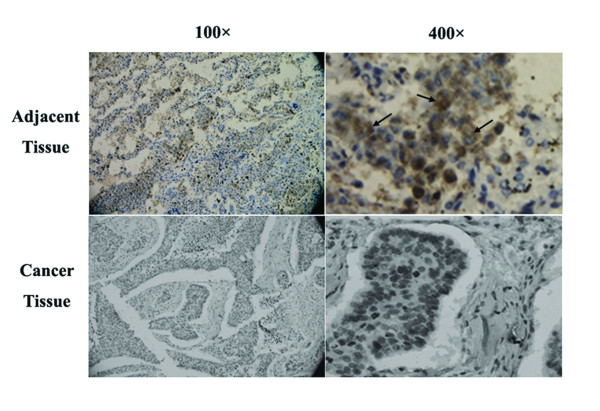
**Immunohistochemical staining of MTSS1 in human oesophageal tissues**. Top panel: the MTSS1 protein was found to be stained in the normal oesophageal epithelial cells (indicated by black arrows); Bottom panel: staining of oesophageal cancer cells for MTSS1 was found to be negative in the oesophageal tumour tissues.

To assess the expression pattern of MTSS1 at the protein level, we performed immunohistochemical analysis of MTSS1 in the human oesophageal squamous cell carcinoma tissue sections (n = 35 pairs). Using a specific anti-MTSS1 monoclonal antibody, MTSS1 was detected both in the cytoplasm and nuclei of non-tumour cells (Figure 2-left panels). Upon the analysis of oesophageal tumour tissues we found that the expression levels of MTSS1 were significantly reduced or absent than those in nontumour tissues (Figure 2-right panels). No obvious staining of MTSS1 was observed in stromal cells in either normal or tumour tissues.

### Expression pattern of MTSS1 in oesophageal cancer cell lines

**Figure 3:**
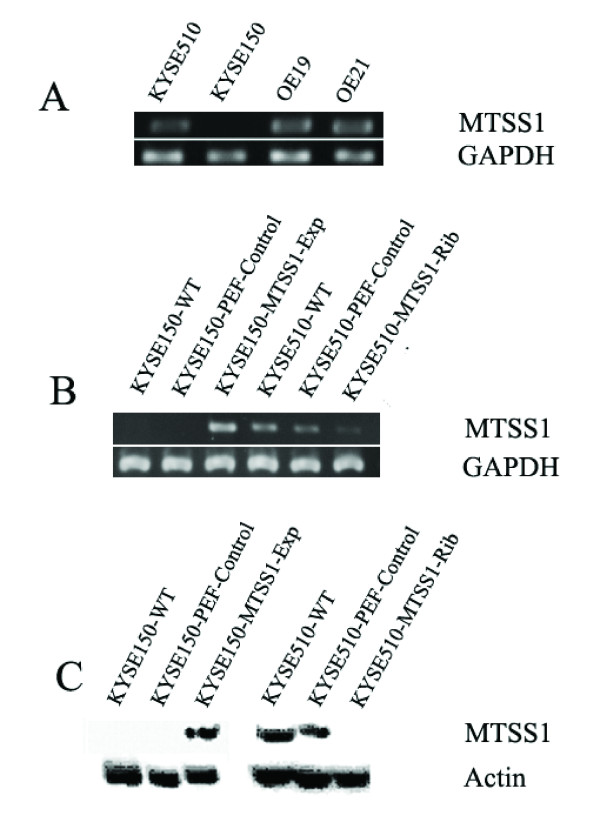
**MTSS1 expression in oesophageal cancer cell lines and over-expression and knockdown of MTSS1**. (A) RT-PCR analysis of MTSS1 mRNA expression within a panel of oesophageal cancer cell lines. MTSS1 was not expressed in KYSE150 oesophageal squamous cell carcinoma cells, but expressed in other three types of oesophageal cancer cell lines. (B) Verification of over-expression and knockdown of MTSS1 in the oesophageal cancer cell lines using RT-PCR. MTSS1 was over-expressed in the KYSE150-MTSS1-Exp cells; whereas MTSS1 expression levels were reduced in the KYSE510-MTSS1-Rib cells. (C) Western blotting confirmation of MTSS1 protein level.

A panel of oesophageal cancer cell lines was examined for the presence of MTSS1 through RT-PCR. MTSS1 transcript was detectable in three cell lines (well or moderate differentiated), but not expressed in the poorly differentiated oesophageal squamous cell carcinoma cell KYSE150 (Figure 3A).

### Stable over-expression and knockdown of MTSS1

To investigate the role of MTSS1 in oesphageal cancer we used KYSE150 for MTSS1 expression as we demonstrated that the wild-type KYSE150 cell line did not express the MTSS1 mRNA. Whereas knockdown of MTSS1 expression was employed from KYSE510 cell line which expressed moderate expression levels of MTSS1. MTSS1 over-expression was successfully established in KYSE150 cells (KYSE150-MTSS1-Exp) after transfection compared with that in KYSE150-WT (KYSE150-wild-type) and empty vector control (KYSE150-PEF-control) cells (Figure 3B). Likewise, MTSS1 which was present within the wild-type and control KYSE510 cells was reduced in the KYSE510-MTSS1-Rib cells. These experiments were replicated at the protein level through Western blotting (Figure 3C). These new MTSS1-modified cell lines were ready for the analysis through a series of *in vitro *studies.

### Regulation of MTSS1 expression had an impact on oesophageal squamous cell carcinoma cell aggressiveness

#### Effects of MTSS1 over-expression or knockdown on in vitro cell growth

**Figure 4:**
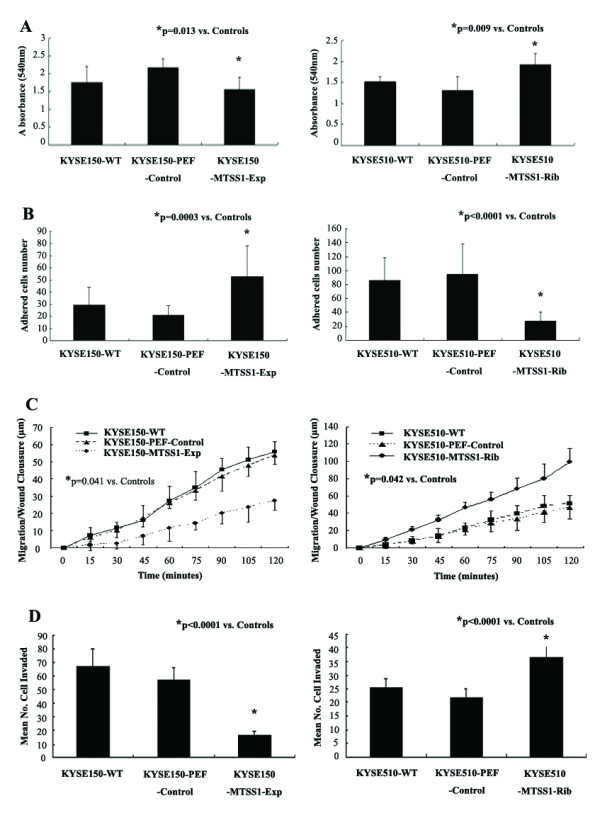
**Cellular function tests of MTSS1 in oesophageal cancer cell lines**. (A) *In vitro *cell growth assay. (Left panel) Over-expression of MTSS1 significantly reduced cell growth rate. (Right panel) Suppression of MTSS1 expression levels enhanced cell growth rate. (B) *In vitro *cell matrix adhesion assay. (Left panel) Cell adhesive ability was dramatically enhanced through over-expression MTSS1 in KYSE150. (Right panel) A reduction in the adhesive nature of KYSE510 was observed through the knockdown of MTSS1 expression. (C) *In vitro *motility assay. (Left panel) Over-expression of MTSS1 significantly inhibited the motile nature of oesophageal cancer cells. (Right panel) Knockdown of MTSS1 expression dramatically enhanced cell migration. (D) *In vitro *invasion. (Left panel) The presence of MTSS1 within KYSE150 significantly suppressed the invasive capacity. (Right panel) Knockdown of MTSS1 promoted the invasiveness of KYSE510. Each cell line was tested in triplicate, three independent experiments were done. Bars, SD.

We first determined the effect of MTSS1 over-expression on *in vitro *cell growth (Figure 4A). The results suggest an inhibitory effect on cell growth by MTSS1 over-expression in oesphageal cancer cells. KYSE150-MTSS1-Exp oesophageal cancer cells had a minor yet significantly reduced rate of growth (p = 0.013) compared to the control group. This was consistent with observations in KYSE510-MTSS1-Rib cells, in which MTSS1 expression had been knocked down. The increased rate of growth (p = 0.009) compared to the control group was seen in KYSE510-MTSS1-Rib cells.

#### Effects of MTSS1 over-expression or knockdown on in vitro cell matrix adhesion

We further examined the influence of MTSS1 on the adhesive nature of these oesophageal cancer cells (Figure 4B). Over-expressing MTSS1 in KYSE150 significantly enhanced the adhesive properties compared to the control group (p = 0.0003). Conversely, knockdown of MTSS1 expression resulted a dramatic reduction in adhesive ability (P < 0.0001).

#### Effects of MTSS1 over-expression or knockdown on in vitro motility

In vitro wounding assay was employed to examine the influence of MTSS1 over-expression or knockdown on oesophageal cancer cell biological behavior. Over-expression of MTSS1 also significantly inhibited the motile nature of oesophageal cancer cells (Figure 4C). The presence of MTSS1 within the cells significantly suppressed cell migration to close the wound compared to the controls (p < 0.05). The result was also consistent with observations in MTSS1 knockdown cells. Cell migration was enhanced in KYSE510-MTSS1-Rib cells compared to the control group (p < 0.05).

#### Effects of MTSS1 over-expression or knockdown on in vitro invasion

Finally, the presence of MTSS1 has also been shown to affect oesophageal cancer cells invasion (Figure 4D). Over-expression of MTSS1 in KYSE150 resulted in a dramatic reduction in the degree of invasion (p < 0.0001 versus controls). This was also confirmed by further determination on the invasive nature of MTSS1 knockdown cells. KYSE510-MTSS1-Rib oesophageal cancer cells were significantly more invasive than the control cells which expressed MTSS1 (p < 0.0001).

## Discussion

Since the study of MTSS1 has been restricted to a limited number of cancer types and available data seem to be controversial, whether or not MTSS1 serves as a metastasis suppressor has not been clearly defined to date. Several lines of evidence have indicated that the expression of MTSS1 could be down-regulated in solid tumours [2,12,15,16], whereas up-regulation of MTSS1 expression has also been observed in one other tumour type [3]. Thus. the role of MTSS1 in cancer and cancer metastasis remains somewhat open. To our best knowledge, the current study is the first report of down-regulation of MTSS1 in oesophageal squamous cell carcinoma. Our study has shown a reduced or absent levels of MTSS1 both in oesophageal squamous cell carcinoma tumour tissues and cancer cell line. We also reported that the expression of MTSS1 was associated with the clinical pathology and prognosis of the patients with oesophageal squamous cell carcinoma. Cellular function tests further demonstrated that the presence of MTSS1 is related to the inhibition of the oesophageal squamous cell carcinoma cell aggressiveness.

MTSS1 has been found to be transcriptionally expressed at lower levels or absent in a limited number of tumour cells. In the present study, the expression levels of MTSS1 were examined in several oesophageal cancer cell lines with different aggressiveness (from well or moderate to poorly differentiated). It is evident from the present study that MTSS1 was absent only in a poorly differentiated cell line, but was maintained in the well or moderate differentiated cell lines, which indicated that MTSS1 expression may be associated with more aggressive cell lines within the same type of cancer.

Perhaps the most important observation in the present study is the relationship between MTSS1 expression and oesophageal squamous cell carcinoma patient clinical data in a cohort of human oesophageal squamous cell carcinoma specimens by using quantitative PCR and immunohistochemical analysis. Our data demonstrated a reduced level of MTSS1 expression in oesophageal squamous cell carcinoma tumours compared to the normal tissues. This in contrary to the results obtained in hepatocellular carcinoma, but clearly in line with the studies in other types of cancer to date. A highly significant link was also seen between MTSS1 expression and nodal status, tumour grade and overall survival. Our findings clearly indicate therefore that MTSS1 may serve as a potential prognostic indicator for patients with oesophageal squamous cell carcinoma, as we show that patients expressing high levels of MTSS1 have a favorable prognosis in contrast to those patients with reduced levels of MTSS1 and a poor prognosis. Consistent with our findings, high levels of MTSS1 expression was also found in breast cancer or hepatocellular carcinoma patients with a favorable prognosis in the previous reports. This indicates that MTSS1 serves as a potential prognostic indicator in human cancer.

Our functional studies have demonstrated that the MTSS1 over-expression resulted in a dramatic reduction in tumour cell migration, invasion and growth, and an increase in cell adhesion. The loss of MTSS1 by way of hammerhead ribozyme transgenes resulted in enhanced invasiveness, migration, growth and decreased adhesive ability, in comparison with control cells, which further verified the results of MTSS1 expression. The inhibition effect of MTSS1 on oesophageal cell growth is in agreement with the findings in prostate cell lines [16,17]. The effect of overexpressed MIM on Shh signaling may be involved in the mechanism for growth suppression [8]. Studies also reported that MIM regulates cell motility by modulating actin polymerization factors through different signaling pathway [4,7,8,18-23], although the detailed mechanism of MTSS1's effect on cell motility need to be further defined. It is interesting to note the enhanced adhesive ability induced by MTSS1, which was in contrast to the findings that MTSS1 did not affect the ability of cell adhesion [16,17]. The mechanism of MTSS1's effect on cell adhesion properties may be related to the role of MTSS1 in cell polarity. Mattila *et al*. suggested that MIM may help promote and maintain cell polarity, whereas the loss of polarity in epithelial cells may affect adhesion [6]. Recent study revealed that MTSS1 deficient mice display defects in the intercellular junctions of epithelial cells. Thus, MTSS1 appears to contribute to the integrity of epithelial sheets, which may provide an explanation for why the loss of MTSS1 in certain epithelial tumours is linked to increased metastatic behaviour [24,25].

## Conclusions

Down-regulation of MTSS1 expression was observed both in oesophageal tumour tissues and oesophageal squamous cell carcinoma cancer cell lines, and is related to clinical pathology and prognosis of the patients with oesophageal squamous cell carcinoma. Cellular function tests further demonstrated that the presence of MTSS1 is related to the inhibition of the oesophageal squamous cell carcinoma cell aggressiveness. This study showed that MTSS1 could be of value as a potential prognostic indicator in human ESCC and may be an important target for cancer therapy.

## Competing interests

The authors declare that they have no competing interests.

## Authors' contributions

FX carried out the Quantitative PCR, western blotting, cell function test, and drafted the manuscript. YL carried out the RNA extraction, reverse transcription PCR, immunoassays and performed the statistical analysis. LY carried out the construction of MTSS1 expressing and ribozyme transgenes. JC participated in the collection of tissue samples and investigated the clinical features. NW participated in collection of the tissue samples. ZZ helped to draft the manuscript. YY participated in the design of the study. LZ participated in the design of the study. WJ conceived of the study, and participated in its design and coordination. All authors read and approved the final manuscript.
